# A Real Time Method for Distinguishing COVID-19 Utilizing 2D-CNN and Transfer Learning

**DOI:** 10.3390/s23094458

**Published:** 2023-05-03

**Authors:** Abida Sultana, Md. Nahiduzzaman, Sagor Chandro Bakchy, Saleh Mohammed Shahriar, Hasibul Islam Peyal, Muhammad E. H. Chowdhury, Amith Khandakar, Mohamed Arselene Ayari, Mominul Ahsan, Julfikar Haider

**Affiliations:** 1 Department of Electrical & Computer Engineering, Rajshahi University of Engineering & Technology, Rajshahi 6204, Bangladesh; 2 Department of Electrical Engineering, Qatar University, Doha 2713, Qatar; 3 Department of Civil and Architectural Engineering, Qatar University, Doha 2713, Qatar; 4 Department of Computer Science, University of York, Deramore Lane, Heslington, York YO10 5GH, UK; 5 Department of Engineering, Manchester Metropolitan University, Chester Street, Manchester M1 5GD, UK

**Keywords:** COVID-19, viral pneumonia, bacterial pneumonia, fibrosis, tuberculosis, pre-trained CNN, chest X-ray

## Abstract

Rapid identification of COVID-19 can assist in making decisions for effective treatment and epidemic prevention. The PCR-based test is expert-dependent, is time-consuming, and has limited sensitivity. By inspecting Chest R-ray (CXR) images, COVID-19, pneumonia, and other lung infections can be detected in real time. The current, state-of-the-art literature suggests that deep learning (DL) is highly advantageous in automatic disease classification utilizing the CXR images. The goal of this study is to develop models by employing DL models for identifying COVID-19 and other lung disorders more efficiently. For this study, a dataset of 18,564 CXR images with seven disease categories was created from multiple publicly available sources. Four DL architectures including the proposed CNN model and pretrained VGG-16, VGG-19, and Inception-v3 models were applied to identify healthy and six lung diseases (fibrosis, lung opacity, viral pneumonia, bacterial pneumonia, COVID-19, and tuberculosis). Accuracy, precision, recall, f1 score, area under the curve (AUC), and testing time were used to evaluate the performance of these four models. The results demonstrated that the proposed CNN model outperformed all other DL models employed for a seven-class classification with an accuracy of 93.15% and average values for precision, recall, f1-score, and AUC of 0.9343, 0.9443, 0.9386, and 0.9939. The CNN model equally performed well when other multiclass classifications including normal and COVID-19 as the common classes were considered, yielding accuracy values of 98%, 97.49%, 97.81%, 96%, and 96.75% for two, three, four, five, and six classes, respectively. The proposed model can also identify COVID-19 with shorter training and testing times compared to other transfer learning models.

## 1. Introduction

Extreme causes of respiratory disorder induce Corona virus disease 2019 (COVID-19), a worldwide pandemic that is still spreading. This new virus was first identified in an outbreak in Wuhan, China, in December 2019 [1]. Globally, the current COVID-19 pandemic has caused 754 million cases and nearly 7 million deaths [2]. One of the most pressing concerns confronting the medical and healthcare sectors is the rapid identification of COVID-19 in an inexpensive way. COVID-19 infection is characterized by fever, cough, tiredness, sputum production, headache, hemoptysis, diarrhea, dyspnea, and lymphopenia [3]. Polymerase chain reaction (PCR)-based test is one of the current techniques for affirming the presence of the virus in human body. Unfortunately, such diagnostic tests take a long time and have low sensitivity, with findings consuming a day or two to arrive. Therefore, the longer waiting time without any awareness of the presence of the virus may cause further spreading [4]. Therefore, correct diagnosis within a shorter period of time is absolutely vital to confine the virus.

Other COVID-19 diagnostic methods include positive radiographic imaging such as computed tomography (CT) or chest radiograph (CXR). Chest radiography can help with a variety of issues, including diagnosing infection and finding false-negative PCR samples [5]. CT scan is a procedure related to high radiation levels and typically more expensive compared to the CXR [6]. Radiologists typically use the CXR method as their first line of defense to find respiratory illnesses in a case of limited number of patients [7]. X-ray imaging is the sole focus of this study, which is limited to patients suspected to have COVID-19. However, despite the fact that conventional CXR scans can assist in the early identification of COVID-19 cases, the images of viral pneumonia are identical to and coincide with those of other respiratory related lung diseases. As a result, the radiologists are facing challenges in identifying the difference between COVID-19 and viral pneumonia or other lung diseases.

Artificial intelligence utilizing DL algorithms in medical imaging could provide a quick, low-cost, and reliable technique to contribute to the medical assessment of COVID-19 [8,9]. Numerous studies have been conducted on computer vision-based early identification of COVID-19 as well as other disorders from the CXR images. For example, the study proposed in [10] provides image processing-based disease detection of pneumonia utilizing the DL approaches. Furthermore, various other researchers have utilized DL networks to identify diseases in diverse areas, including retinal image inspection [11], lung cancer [12], skin cancer [13], tuberculosis [14], and many more.

Since the outbreak of COVID-19, different attempts have been tried with the CXR images to diagnosis COVID-19 infection. However, because of two major problems, this sector has not made much progress in recognizing COVID-19 infection for practical use. The first problem is the lack of relevant data availability whereas the lack of standardized methods and limited disease classes with appropriate annotations are the second problem. Motivated by these finding, the authors have combined a variety of databases for creating a large database and developing a classification scheme that includes seven categories: COVID-19, Normal, Viral Pneumonia, Bacterial Pneumonia, Lung Opacity, Fibrosis, and Tuberculosis. In this study, larger dataset is used to train models that can offer quick and accurate recognition of COVID-19. A database is created from 18,564 CXR images by combining multiple databases, with seven classes (i.e., Normal, COVID-19, Viral Pneumonia, Bacterial Pneumonia, Fibrosis, Lung Opacity, and Tuberculosis). The performance of seven-class classifications has been investigated using a proposed convolutional neural network (CNN) model and compared with three pre-trained DL networks (VGG-16, VGG-19, and Inception-v3 model).

However, sometimes a situation can arrive when all seven classes are not required to detect. Then, some of the classes can be eliminated to improve the accuracy. This prompts to develop models for the classification of binary to small number classes. In the first case, a binary CXR database with two classes—Normal and COVID-19—was used. By keeping these two classes as the common ones, four other cases were developed upon adding one new class in a cumulative manner where the last case included all seven disease classes. Furthermore, three pre-trained networks (VGG-16, VGG-19, and Inception-v3 models) are compared with the proposed CNN architecture.

The significance of the proposed CNN model lies in its ability to accurately classify COVID-19 and multiple other lung diseases with lower testing time. This is achieved by training the model on a combined dataset created from three separate datasets. The proposed model outperforms pre-trained models in terms of speed and accuracy, making it a useful tool for the detection of lung-related diseases in real-world settings. The model’s reliability has been confirmed through experiments with two to six classes of diseases, further adding to its practical significance.

The key contributions of this paper include:

(1) A larger dataset of CXR images with seven classes (COVID-19, Normal, Viral Pneumonia, Bacterial Pneumonia, Fibrosis, Lung Opacity, and Tuberculosis) is developed by combining several small datasets to create a real life multiclass problem.
(2) The imbalanced dataset is transformed into a balanced dataset for training by applying augmentation to improve the model’s performance.
(3) The proposed CNN and three pre-trained CNN models (VGG-16, VGG-19, and Inception-v3 model) have been developed to detect COVID-19 and other lung disorders from the CXR images for various classification tasks.
(4) The proposed CNN model is compared with the pretrained models on classification performance and processing speed.

The following is an outline of the sections of the paper: Section 2 is composed of studies related to this subject. Section 3 explains the suggested methodology of the strategy, including the database description, algorithms, and metrics used to evaluate the technique. In Section 4, the experimental analysis and results are given. In Section 5, a comparative literature review is presented. A discussion of the result and future work concludes Section 6.

## 2. Related Works

Recently, a number of researchers have employed various deep learning algorithms to automatically identify COVID-19 and other disorders; the bulk of these methods have shown exceptionally high levels of accuracy. The following COVID-19 detection topic had been the subject of a number of studies in the past [15,16,17]. Hemdan et al. [18] worked on binary classification. In their study, they employed a total of 50 CXR scans, including 25 for COVID and 25 for healthy. COVIDX-Net was proposed as a system based on seven pre-trained models (VGG19, DenseNet201, ResNetV2, InceptionV3, InceptionResNetV2, Xception, and MobileNetV2). In their study, VGG-19 was the best classifier, with an accuracy of 90% and f1 scores of 0.91 and 0.81 for COVID-19 and healthy, respectively, but InceptionV3 was the lowest, with an accuracy of 50% and f1 scores of 0.67 and 0.00 for healthy and COVID-19, respectively. Moreover, 180 COVID-19 and 200 normal CXR images were used in the binary classification experiment by Ismael et al. [19]. Their research yielded a 94.7% accuracy rate using deep features taken from the ResNet50 model and SVM classifier with the linear kernel function. Using CXR images, Ohota et al. [20] proposed COVID-19 detection using two databases with identical COVID-19 category images and distinct healthy images. With a trained CNN as the feature extractor, pretrained CNN models (MobileNet, DenseNet121, DenseNet169, DenseNet201, VGG16, and VGG19) were used with classical machine learning methods. MobileNet architecture combined with SVM (Accuracy: 98.462% and F1-Score: 98.461%) delivered the best performance for one dataset, while DenseNet201 with MLP generated the best performance for another dataset.

CheXNet algorithm, developed by Pranav et al. [21], was used to diagnose pneumonia from the CXR images more precisely in contrast to expert doctors. In their study, CheXNet was trained using the largest available online CXR database, which included about 100,000 X-ray images of 14 diseases. They compared CheXNet’s performance to that of professional radiologists using the F1 metric and found that CheXNet outperformed a typical radiologist’s assessment.

In another study, Rahman et al. [14] employed nine different pre-trained CNN models (ResNet18, ResNet50, ResNet101, ChexNet, InceptionV3, Vgg19, DenseNet201, SqueezeNet, and MobileNet) to arrange CXR images into two categories (tuberculosis and healthy). The ChexNet and DenseNet201 models outperformed in the augmentation of both scenarios (with and without image). Furthermore, Chowdhury et al. [22] created a public database by integrating various other public databases in order to gather data on 423 cases of COVID-19, 1485 cases of viral pneumonia, and 1579 cases of normal CXR. They utilized several CNN models that were pre-trained to categorize CXR images of two different approaches: (1) Normal and COVID-19 cases; (2) Normal, viral pneumonia, and COVID-19 cases, with and without image augmentation. Cannata et al. [23] used the Vision Transformer and three transfer learning models (Inception-v3, ResNet50, and Xception) to recognize four different states from CXR images (normal, viral pneumonia, lung opacity, and COVID-19). The Vision Transformer obtained a 99.3% accuracy over the convolutional architecture. Brunese et al. [24] employed 6523 CXR images to test a transfer learning-based VGG-16 model for COVID-19 detection, including 250 COVID-19 patients, 2753 patients with pulmonary diseases, and 3520 healthy patients. Based on the VGG-16, two models were developed: (1) to discriminate between healthy patients and patients with COVID-19 and pulmonary disorders with 96% accuracy and (2) to characterize COVID-19 patients and patients with pulmonary diseases with 98% accuracy. Ozturk et al. [25] introduced a DarkCovidNet model for accurate COVID-19 identification. Their technique achieved 98.08% accuracy for binary (COVID-19 and Non-COVID-19) classifications and 87.0% accuracy for multi-class (COVID-19, Non-COVID-19, and Pneumonia) classifications. Chandra et al. [26] established an effective COVID-19 test that included 2088 CXR images (696 normal, 696 pneumonia, and 696 COVID-19), as well as 258 (86 images of each class) images and had an accuracy of 98%. Vantaggiato et al. [27] proposed a three-class case and a five-class case for distinguishing COVID-19 scans from other lung disorders in another investigation. They utilized Ensemble-CNN, ResNeXt-50, Inception v3, DenseNet-161, and ResNeXt-50 for these cases. In their study, Ensemble-CNN performed admirably in detecting COVID-19 infections in three-class and five-class circumstances, with corresponding accuracies of 100% and 98.1%. Additionally, Nahiduzzaman et al. [28] developed ChestX-ray6, a CNN that could promptly and accurately detect four distinct illnesses, including COVID-19 and a healthy sample. Using the ChestX-ray6 model, five distinct diseases, including those without disease, were recognized with an accuracy of 80%. In addition, the scientists evaluated their pre-trained ChestX-Ray6 model using a binary class dataset containing normal and pneumonia cases, achieving an accuracy of 97.94% and a recall of 98%.

Khan et al. [29] utilized a dataset of 270 patients, of which 48 were healthy and 222 were COVID-19-positive. After that, a fusion method was developed that improves diagnostic precision by combining CXR data with clinical information. Using clinical data and the CXR, a deep learning model was created with an accuracy of 97.0%, a recall of 0.986, a precision of 0.978, and an F1-score of 0.982. In addition, Umair et al. [30] used the four pretrained models to identify COVID-19 in a normal sample; the accuracy rates for VGG16, ResNet-50, DenseNet-121, and MobileNet were 83.27%, 92.48%, 96.49%, and 96.49%, respectively. In their investigation, class-specific heatmap images were also produced using the Grad-CAM method to further emphasize the features extracted from the X-ray images. Alam et al. [31] suggested an imaging-based fusion method for detecting COVID-19 and healthy samples using CXR images. They merged features extracted from CXR images with HOG and CNN, trained VGG-19, and attained a testing accuracy of 99.49%. In addition, Li et al. [32] created an automated diagnosis system known as Cov-net, which operated with two publicly available databases for diagnosing two-class and three-class problems. In three-category (COVID-19, normal, and viral pneumonia) and four-category (COVID-19, normal, lung opacity, and viral pneumonia) classification issues, their model outperformed the pre-trained model with an accuracy of 99.66% and 96.49%, respectively. Additionally, Babukarthik et al. [33] built a GDCNN model to predict and classify various pneumonia illnesses using CXR images. In their proposed method, they utilized Huddle Particle Swarm Optimization to reach a classification accuracy of 97.23% for a range of pneumonia disorders, including COVID-19. Moreover, a survey was conducted by Shorten et al. [34] concerning the application of deep learning to the COVID-19 pandemic as well as the application of deep learning in many other fields. Additionally, they explained how tasks were modeled as learning problems using Deep Neural Networks and showed how various data types were inputted.

According to the state-of-the-art (SOTA) literature, even though some researchers employed the deep learning technique to recognize COVID-19 using CXR images, the majority produced models with a limited number of images in the dataset for classifying two- to four-class schemes. Due to the relatively small size of the datasets utilized in these studies, it cannot be assured that the suggested models will function with the same precision on a larger dataset containing images from several sources. In addition, when a model is trained using a smaller dataset, model overfitting may occur. Motivated by this conclusion, the authors of this study developed a larger and more balanced dataset by combining multiple smaller datasets with picture enhancement, which included seven classifications of lung disorders.

## 3. Methodologies

**Figure 1:**
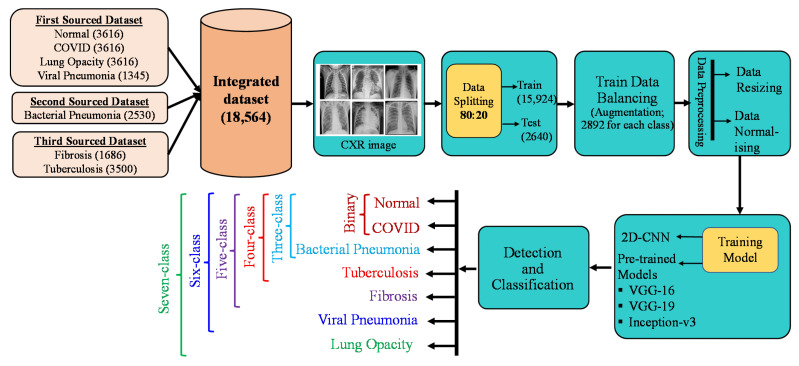
An illustration of COVID-19 detection framework implemented in this study.

Figure 1 depicts the full methodical process for differentiating the COVID-19 class from other lung disease classes as well as a healthy class using CXR images. CXR images from various publicly accessible sources were combined to create a larger dataset with seven classes. The dataset was divided into 80% for training and 20% for testing. The training data was balanced, and then the data were pre-processed. Following that, models for binary, three-class, four-class, five-class, six-class, and seven-class schemes were created utilizing transfer learning techniques and the proposed CNN. Finally, the model’s performance was evaluated using various performance criteria.

### 3.1. Data Collection

Three datasets with CXR images from Kaggle were used in this study.

#### 3.1.1. COVID-19 Radiography Database

A database of CXR images including COVID-19 (219), normal (1341), and viral pneumonia (1345) was built by researchers and academics from Qatar and Bangladesh, among others [35]. During their initial upgrade, 1200 CXR images of the COVID-19 category were included. Further COVID-19 (3616) as well as normal (10,192), lung opacity (6012), and viral pneumonia (1345) CXR images and lung masks were added to the database in the second update. COVID-19, normal, lung opacity, and viral pneumonia images were used in this study from the second update database. The objective of this paper was to separate COVID-19 from other lung disorders. The number of normal and lung opacity images were higher than that of the COVID-19 images; therefore, some images were removed randomly to make the numbers equal (3616) for all three classes. Furthermore, 1345 CXR viral pneumonia images were also utilized in this study.

#### 3.1.2. Viral Pneumonia vs. Bacterial Pneumonia Database

Kaggle provided the 4273 CXR image dataset where there were 1493 for Pneumonia (Virus) and 2780 for Pneumonia (Bacteria) [36,37]. From this database, 2530 images for bacterial pneumonia were only used for this study. 

#### 3.1.3. CXR Database for Tuberculosis (TB) and Fibrosis

The CXR image dataset for COVID-19, normal, tuberculosis, fibrosis, and pneumonia are made available in Kaggle from publicly available dataset [14,38]. Only tuberculosis (3500) and fibrosis (1686) images were extracted from this database.

#### 3.1.4. New Dataset Creation

**Figure 2:**
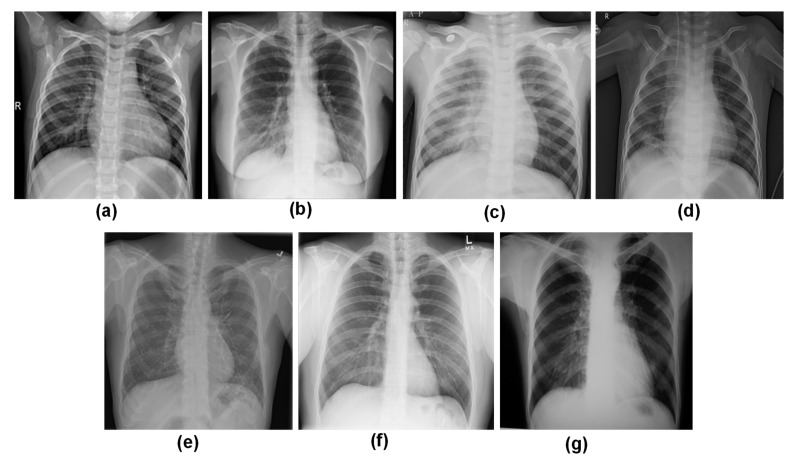
Representation of CXR images from the developed dataset: (**a**) Normal, (**b**) COVID, (**c**) Viral Pneumonia, (**d**) Bacterial Pneumonia, (**e**) Fibrosis, (**f**) Lung Opacity, and (**g**) Tuberculosis.

Finally, a new dataset was created for this study by integrating the three individual datasets. The new dataset consists of 18,564 CXR images with seven classes. Figure 2 depicts example CXR images in the dataset for different classes. Individual dataset characteristics and the new dataset creation method are described in Table 1.

### 3.2. Dataset Splitting

The images in the dataset were randomly divided into training (15,924) and testing (2640) sets with a ratio of 80:20 for each class. Table 2 represents the proportion of the number of images for the training and testing sets.

### 3.3. Training Data Balancing

When the training data is imbalanced, the model frequently overclassifies the majority group due to its greater prior probability. As a result, minority cases are misclassified at a higher rate than majority instances [39]. Data balance prevents a dataset from getting biased towards a specific class during model training. As a result, even if more data are added to train the model, it will not be biased in favor of the majority group. The training data in the new dataset were skewed for the bacterial pneumonia, viral pneumonia, tuberculosis, and fibrosis classes. The data augmentation strategy was utilized in this work to create balanced training data from unbalanced ones by increasing the number of images. This study includes a total of 20,244 CXR images after augmentation, including 2892 images in each class.

**Figure 3:**
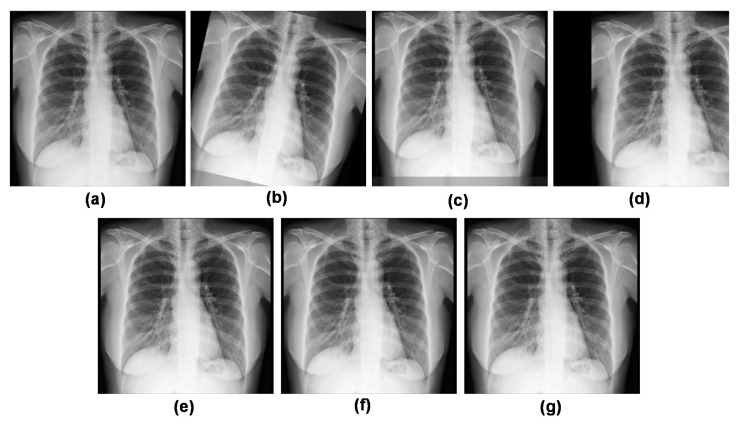
Example images after augmentation: (**a**) Original, (**b**) Rotation (30°), (**c**) Width Shift (20%), (**d**) Height Shift (20%) (**e**) Shearing (20%), (**f**) Zooming (20%), (**g**) Fill Mode (Nearest).

This study employed six augmentation techniques as shown in Figure 3. A rotation range of 30 degrees was utilized to rotate the image at random between 0 and 30 degrees. The second and third parameters (width shift range = 0.2 and height shift range = 0.2, respectively) moved the images by 20% in both the horizontal and vertical directions. With a shear range of 0.2, the fourth parameter sheared the images by 20%. The fifth option used a 20% zoom range to zoom in and out of the images. The last augmentation involved expanding the region and filling it with the closest pixel.

### 3.4. Data Pre-Processing

Because neural networks generally receive images of equal size, scaling images is an important pre-processing step in computer vision [40]. All images are shrunk to indistinguishable sizes before being fed into the CNN. The CXR images from the balanced database were downsized to a fixed size of 299 × 299 pixels in this investigation without modifying the file formats.

Images are stored in a computer as a matrix of integers known as pixel values. These pixel values represent the intensity of each pixel, with 0 representing black and 255 representing white. If the as-received image is used to train a deep neural network, the computation of huge numeric pixel values may become increasingly difficult. Normalization is an image processing technique that modifies the range of pixel intensity values [41]. To reduce computational difficulties, pixel values were normalized to a range of 0 to 1 by dividing them by 255.

### 3.5. Deep learning Architectures

#### 3.5.1. Proposed 2D-CNN Architecture

**Figure 4:**
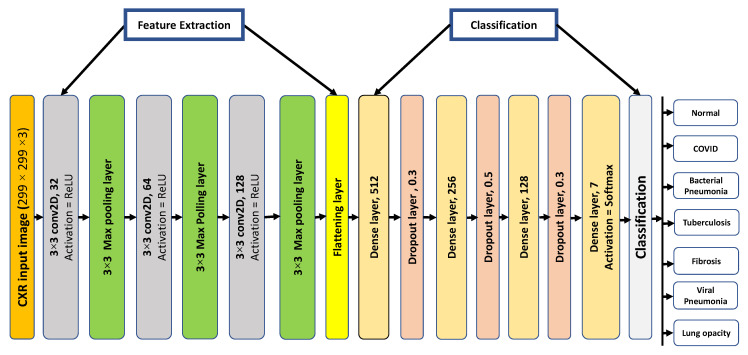
The proposed 2D-CNN model architecture for different classification schemes.

The proposed CNN architecture for COVID-19 classification using the CXR images, along with other lung diseases and healthy subjects, is shown in Figure 4. In this work, two-dimensional convolutions were considered based on 32 filters in the first layer. Following the first convolutional layer, the max-pooling layer with a pool size of 3 × 3 and a stride of 1 was arranged. After the first convolutional layer, the second and third layers were run concurrently with 64 and 128 filters, respectively. The three convolutional layers were constructed with a kernel size of 3 × 3 over a stride of 1, and they are all activated using the ReLu function. Multidimensional feature vectors were presented in the output which were transformed into 2-dimensional feature vectors to be supplied to the fully connected layer, that is performed by the subsequent flattening layer. The transformed output was sent to the dense or fully linked layer of 512 units. Afterward, a dropout layer, a subsequent dense layer of 256 units, a dropout layer, a subsequent dense layer of 128 units, and a further dropout layer were added. On the final layer, SoftMax activation was used, and data classification resulted in an output of size equal to class number in different classification schemes. The proposed CNN model was also used in this work for various classification schemes such as binary class, 3-class, 4-class, 5-class, 6-class, and 7-class. The summary of the proposed CNN model is shown in Table 3. The motivation for this proposed model was to achieve better performance than the previously proposed models. By using a trial-and-error methodology, the model’s parameters, including the number of convolutional, dense, and dropout layers and the number of units in each layer, were established. The proposed model’s architecture was modified until the proposed model outperformed the pre-trained models.

#### 3.5.2. Pre-Trained Models Architecture

**Figure 5:**
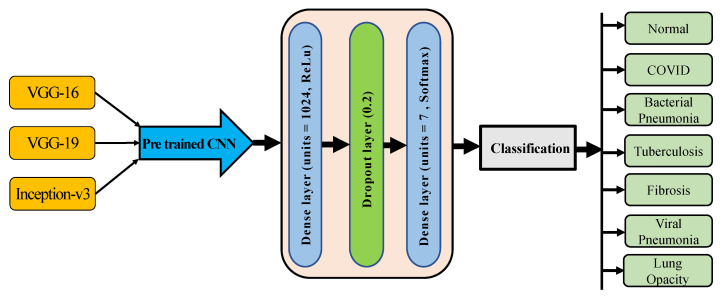
The pretrained model’s architecture for seven classes.

In this work, three pre-trained models were tested. The weights of all layers were changed from trainable to non-trainable as the main concept of transfer learning is to use the information of the previously trained model in a new task. The weights of the pre-trained models were frozen before flattening the layer. The fully connected layers were added after every transfer learning model according to the number of output classes. The dense layer of the pre-trained models was substituted by three additional layers. To prevent overfitting, another 20% dropout layer was added after the first dense layer of 1024 units, and the final output layer was supplied to the SoftMax activation layer, with a size of the output set to 7 (for 7-class scheme). The architecture of the pre-trained models is depicted in Figure 5. The main concern of determining the hyperparameter was to maintain the high performance of both proposed and pretrained models. Trial-and-error methodologies were used to select parameters for the proposed model and pretrained model. The number of parameters of the applied models is shown in Table 4.

### 3.6. Experiment Setup

The proposed CNN and three pre-trained CNN models are constructed utilizing Tensorflow and the Python wrapper library, KERAS. The experiments were conducted on Google Colab (The project Link: https://github.com/akhi16/Lung-Diseases-Detection (accessed on 20 April 2023). The Adam optimizer algorithm was used to optimize the hyperparameters for the proposed CNN and pre-trained CNN models. Categorical-cross-entropy was employed as the loss function, which enabled assessing the network’s error during training. Batch sizes of 64 were used, and dropout rates ranged from 0.2% to 0.5%. The accuracy of the proposed model was tested with various epoch counts, and it was found that after 25 epochs, there was no discernible improvement in accuracy. To demonstrate how the pre-trained models operated, they were likewise trained over the same number of epochs as the proposed model. In the pre-trained models, the maximum accuracy was attained prior to 25 epochs. Various approaches have been analyzed in this study. First, three previously trained models and the proposed CNN model are used to examine seven categorization schemes where the effectiveness of them has been studied. The experimentation result with the two-class, three-class, four-class, five-class, and six-class schemes is also described in this section of the study, applying the proposed CNN.

### 3.7. Evaluation Criteria

In this work, the performance of the proposed CNN and three pre-trained CNN models was evaluated using accuracy, precision, recall, F1 score, and AUC. Accuracy is defined as the number of outputs from a model that are exactly predicted. Precision is a statistic that counts the number of correct positive forecasts produced. A model’s recall is a measure of how well it can recognize True Positives. The F1 score is an evaluation of a model’s precision and recall that takes the model’s correctness into account. Equations (1)–(4) were used to calculate accuracy, precision, recall, and F1-score. In addition, the models’ training and testing periods were documented.

(1) $$\mathrm{Accuracy}\;=\frac{T_{P}+T_{n}}{T_{P}+T_{n}+F_{p}+F_{n}}$$

(2) $$\mathrm{Precision}\;=\frac{T_{P}}{T_{P}+F_{p}}$$

(3) $$\mathrm{Recall}\;=\frac{T_{P}}{T_{P}+F_{n}}$$

(4) $$F1-\mathrm{score}\;=\frac{2*\mathrm{Precision}*\mathrm{Recall}}{\mathrm{Precision}+\mathrm{Recall}}$$

where *T_p_* is True Positive; *T_n_* is True Negative; *F_p_* is False Positive; and *F_n_* is False Negative.

## 4. Experimental Results

### 4.1. Seven-Class Classification System

**Figure 6:**
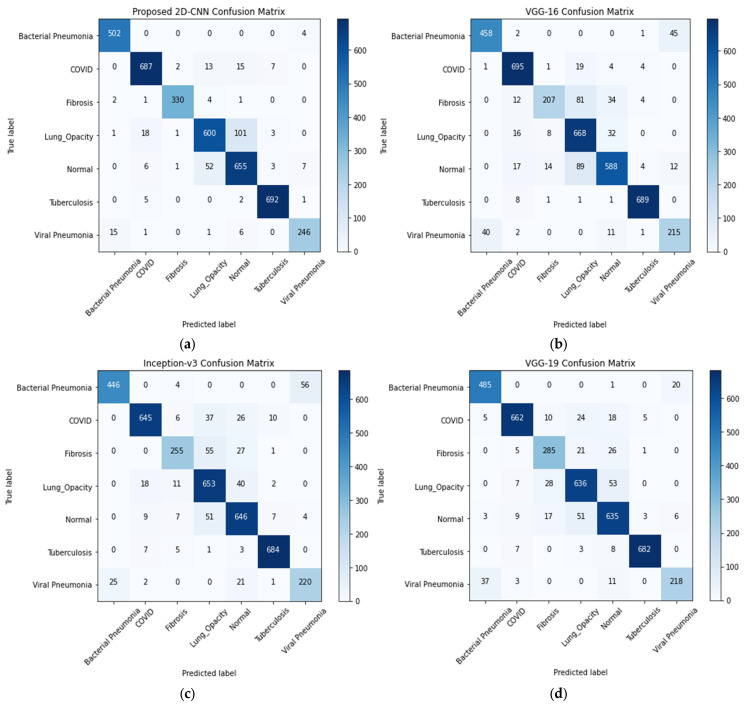
Confusion matrix for (**a**) 2D-CNN, (**b**) VGG-16, (**c**) Inception-v3, and (**d**) VGG-19 models.

The performance of 2D-CNN and other transfer-learning models for seven-class classification scheme is evaluated using a confusion matrix shown in Figure 6. With the proposed CNN model, 502 images were correctly classified, but four images are incorrectly classified for the bacterial pneumonia class. On the other hand, the number of incorrectly classified images for COVID-19, fibrosis, lung opacity, normal, tuberculosis, and viral pneumonia were 37, 8, 124, 69, 8, and 23, respectively. The correctly identified images by the other incorrectly models were lower than that by the proposed model except for the lung opacity class suggesting its improved performance in classifying the lung diseases. However, VGG-16 correctly identified slightly a greater number of images than that of the proposed model.

**Figure 7:**
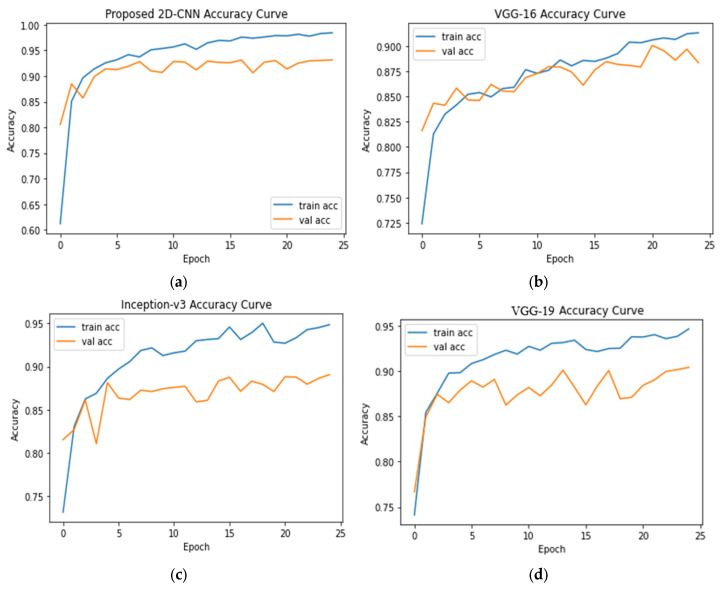
Accuracy curves for (**a**) 2D-CNN, (**b**) VGG-16, (**c**) Inception-v3, and (**d**) VGG-19 models.

Training and testing accuracy curves of the models are shown in Figure 7. The proposed 2D-CNN model’s testing accuracy was approximately 93.15% while the testing accuracies of VGG-16, Inception-v3, and VGG-19 were 88.33%, 89.06%, and 90.41%, respectively.

**Figure 8:**
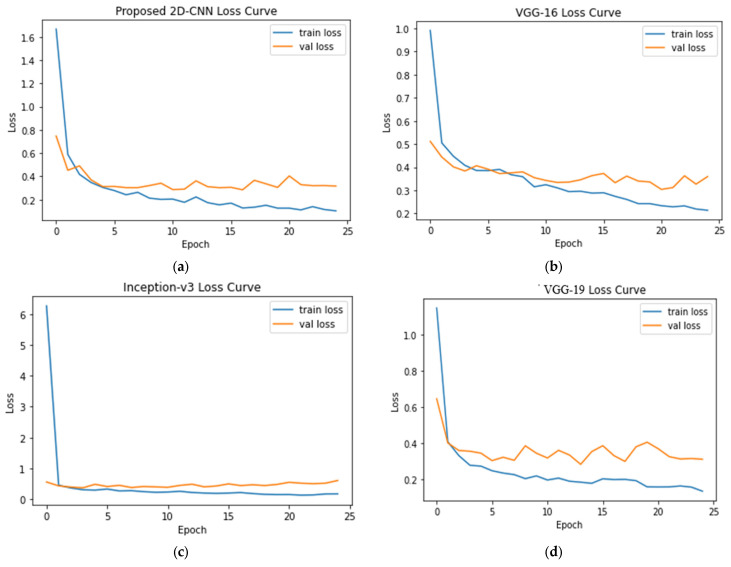
Loss curves for (**a**) 2D-CNN, (**b**) VGG-16, (**c**) Inception-v3, and (**d**) VGG-19 models.

Figure 8 displays the train and testing loss curves for the proposed CNN, VGG-16, VGG-19, and Inceptionv3 models with loss values of 0.3176, 0.3595, 0.6094, and 0.3119, respectively. 

**Figure 9:**
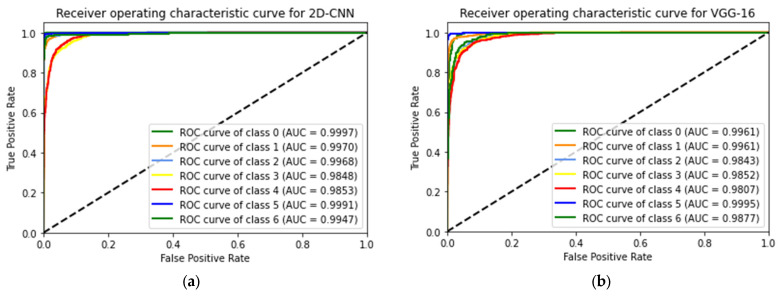

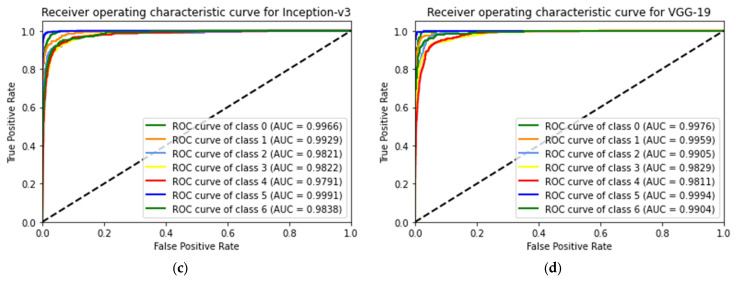
ROC curves for (**a**) 2D-CNN, (**b**) VGG-16, (**c**) Inception-v3, and (**d**) VGG-19 models.

The ROC curves of the four CNN architectures used in this investigation with seven classes are shown in Figure 9. The models exhibit AUC values of 0.98 or higher. For the COVID-19 class, the proposed 2D-CNN achieved an AUC of 0.9970, while VGG-16, Inception-v3, and VGG-19 obtained AUCs of 0.9961, 0.9929, and 0.9959, respectively. Therefore, the proposed CNN produced a higher AUC value for the COVID-19 class than the other three pretrained models.

**Figure 10:**
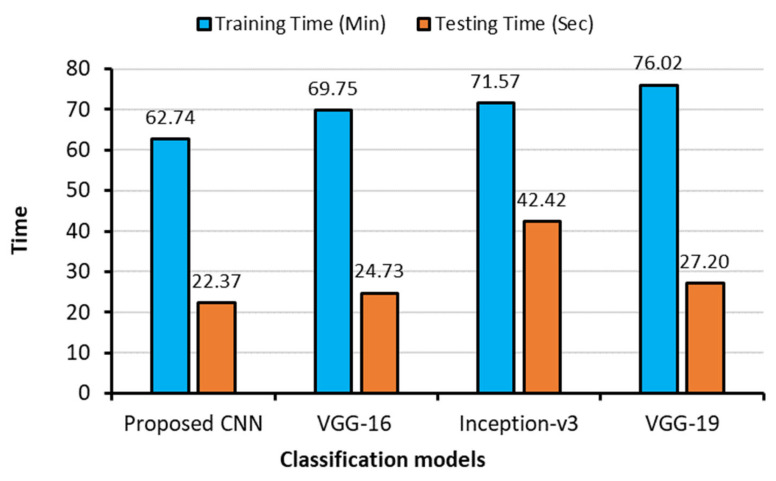
Training and testing times of four deep learning models for the seven-class scheme.

Table 5 depicts the outcomes of four CNN models for recognizing seven classes. The proposed CNN revealed better average accuracy, recall, F1-score, and AUC than the other three CNN models in detecting COVID-19 as well as other lung diseases. VGG-19 and Inception-v3 displayed classification accuracies of 90.41% and 89.06%, respectively, when compared to the proposed CNN model (93.15%). In contrast, the VGG-16 model scores the lowest classification accuracy of 88.33% as shown in Figure 10. The average AUC values for the proposed CNN, VGG-16, VGG-19, and Inception-v3 were, respectively, 0.9939, 0.9899, 0.9879, and 0.9925, demonstrating that the proposed CNN model performed better than the others. When compared the performances for individual classes, the proposed one showed better performances for majority of the classes, but VGG-19 showed better precision for the COVID-19, lung opacity, and tuberculosis, and VGG-19 performed better than the proposed model for the COVID-19 and lung opacity classes in terms of recall and lung opacity and tuberculosis classes in terms of AUC.

**Figure 11:**
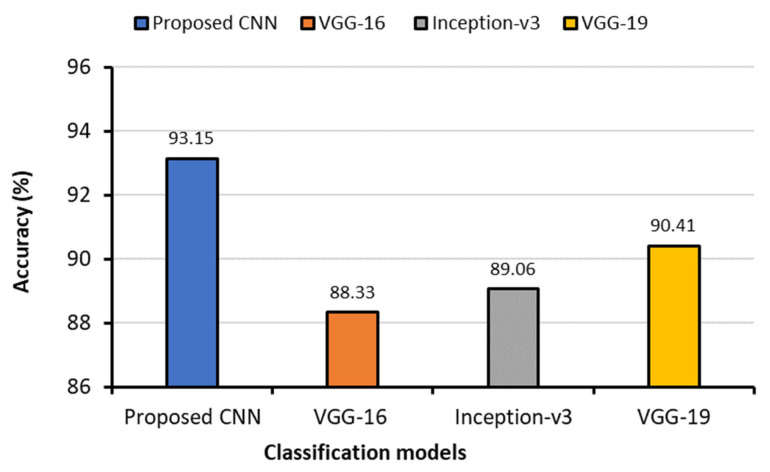
Testing accuracies of different models for the seven-class scheme.

A bar chart illustrating training and testing times is shown in Figure 11. The proposed CNN model took shortest times to train (62.74 min) and test (22.37 s) of all the images available in the training and testing sets. On the other hand, the VGG-19 model spent longest time to train (76.02 min), and Inception-v3 took longest time to test (42.42 s) for all images. Therefore, in terms of testing and training times, the proposed CNN outperformed the other pretrained models.

### 4.2. Six-Class Classification System

**Figure 12:**
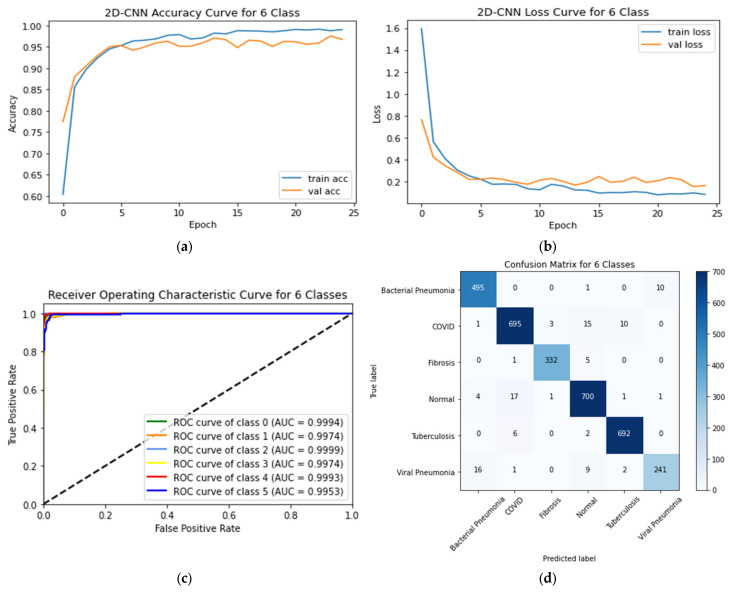
(**a**) Accuracy curves (**b**) loss curves, (**c**) ROC curves, and (**d**) confusion matrix of the proposed CNN models with six individual classes (COVID-19, normal, viral pneumonia, bacterial pneumonia, fibrosis, and tuberculosis).

The performance of the CNN and the transfer models for classifying six classes is shown in Table 6 along with other metrics such as training and testing times. The proposed CNN’s training and testing accuracy curves, ROC curves, and confusion matrix for the six-class scheme are shown in Figure 12. This graph demonstrated that the proposed CNN model achieved a testing accuracy of 96.75%. Six classes can be classified more accurately using the proposed CNN model compared to the seven-class category. The testing loss of the proposed CNN model was 0.1634. The AUC values of all the six classes were greater than 0.99.

### 4.3. Five-Class Classification System

**Figure 13:**
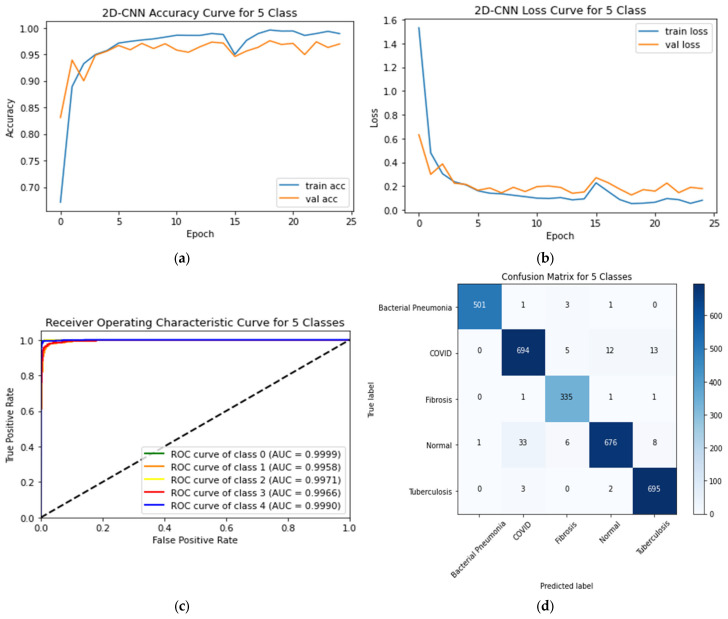
(**a**) Accuracy curves (**b**) loss curves, (**c**) ROC curves, and (**d**) confusion matrix of the proposed 2D-CNN models with five individual classes (Normal, COVID-19, bacterial pneumonia, fibrosis, and tuberculosis).

Table 7 shows performance of the CNN and the transfer learning models for classifying the five-class scheme. The training and testing accuracy curves, ROC curves, and CM of the proposed CNN for the five-class scheme are displayed in Figure 13. From this figure, it is shown that the proposed CNN model has a testing accuracy of 96.96% and a testing loss of 0.1794. The proposed CNN achieved AUC values of 0.9966, 0.9958, 0.9999, 0.9971, and 0.9990 for normal, COVID-19, bacterial pneumonia, fibrosis, and tuberculosis, respectively.

### 4.4. Four-Class Classification System

**Figure 14:**
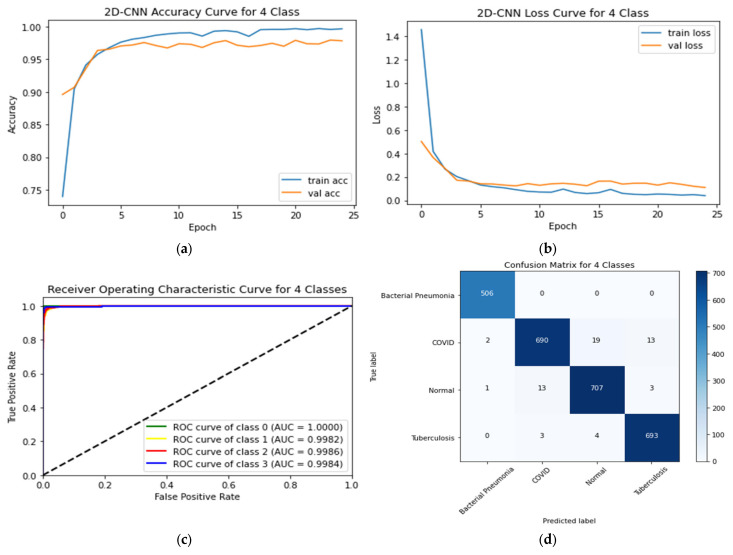
(**a**) Accuracy curves (**b**) loss curves, (**c**) ROC curves, and (**d**) CM of the proposed CNN models with four individual classes (Normal, COVID-19, bacterial pneumonia, and tuberculosis).

Table 8 shows the performance of the proposed CNN, and the transfer learning models for classifying the four-class scheme relating. The training and testing accuracy curves, ROC curves, and CM for the proposed 2D-CNN are illustrated in Figure 14. The proposed CNN model has a testing accuracy of 97.81% and a testing loss of 0.1097. The AUC values for normal, COVID-19, bacterial pneumonia and tuberculosis were 0.9986, 0.9982, 1.0000, and 0.9984, respectively.

### 4.5. Three-Class Classification System

**Figure 15:**
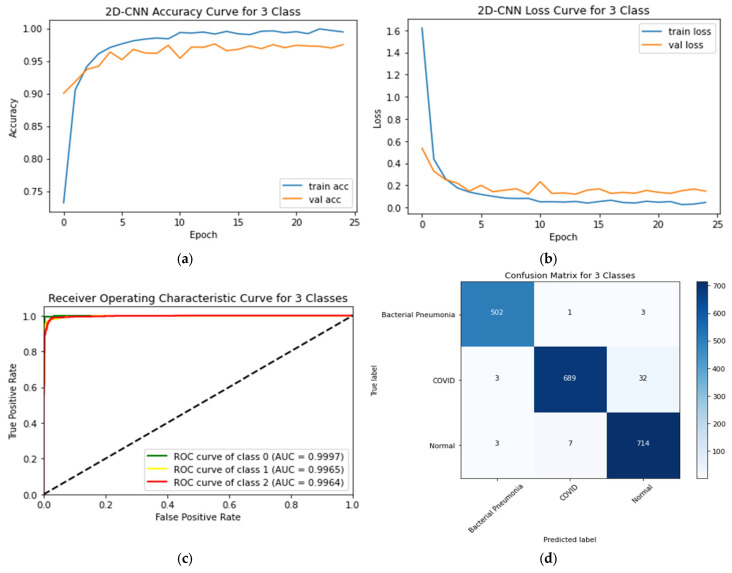
(**a**) Accuracy curves (**b**) loss curves, (**c**) ROC curves, and (**d**) CM of the proposed CNN models with three individual classes (Normal, COVID-19 and bacterial pneumonia).

The outcomes of the three-class case on the testing data are presented in Table 9. Figure 15 depicts the training and testing accuracy curves, ROC curves, and CM for the proposed CNN. This testing accuracy and loss of the proposed CNN model were 97.49% and 0.1473, respectively. The CM indicated that the proposed 2D-CNN model misclassified 32 images as normal and three as bacterial pneumonia during the COVID-19 detection. The AUC values for normal, COVID-19, and bacterial pneumonia were 0.9997, 0.9965, and 0.9997, respectively.

### 4.6. Binary-Class Classification System

**Figure 16:**
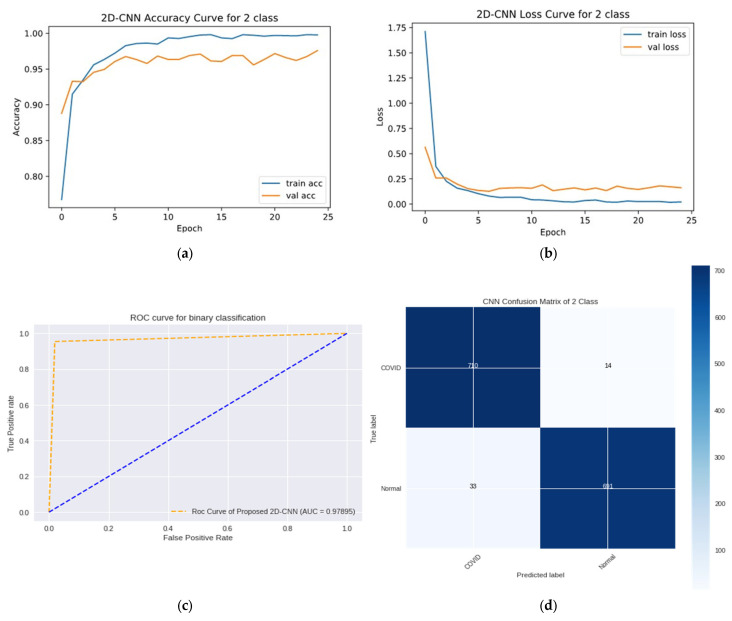
(**a**) Accuracy curves (**b**) loss curves, (**c**) ROC curves, and (**d**) CM of the proposed 2D-CNN models with two individual classes (Normal and COVID-19).

The outcomes of the binary class case on the testing data are presented in Table 10. The results served as the foundation for the proposed CNN model for all the applied assessment criteria (Accuracy, Precision, Recall, F1-score, and AUC). The proposed CNN training and testing accuracy curves, ROC curves, and confusion matrix are shown in Figure 16. This graph illustrates that the proposed 2D-CNN model obtained a testing accuracy 98% and loss of 0.1253. The graph also shows that the proposed 2D-CNN model performed correctly on 98.066% (710 images) of the COVID-19 images and 95.44% (691 images) of the healthy images. The 2D-CNN model misclassified 14 normal images as COVID-19 and misclassified 33 COVID-19 images as normal. The model also achieved an AUC value of 0.9789 for identifying COVID-19 samples compared to the healthy sample. A high recall value of 0.9723 indicates that the model successfully decreased false-negative rates, ensuring that no significant cases of COVID-19 infection were missed. However, the high precision value of 0.9791 shows that the model limited false positive rates; hence, COVID-19 infected cases were not frequently misclassified.

### 4.7. Grad CAM Visualisation

**Figure 17:**
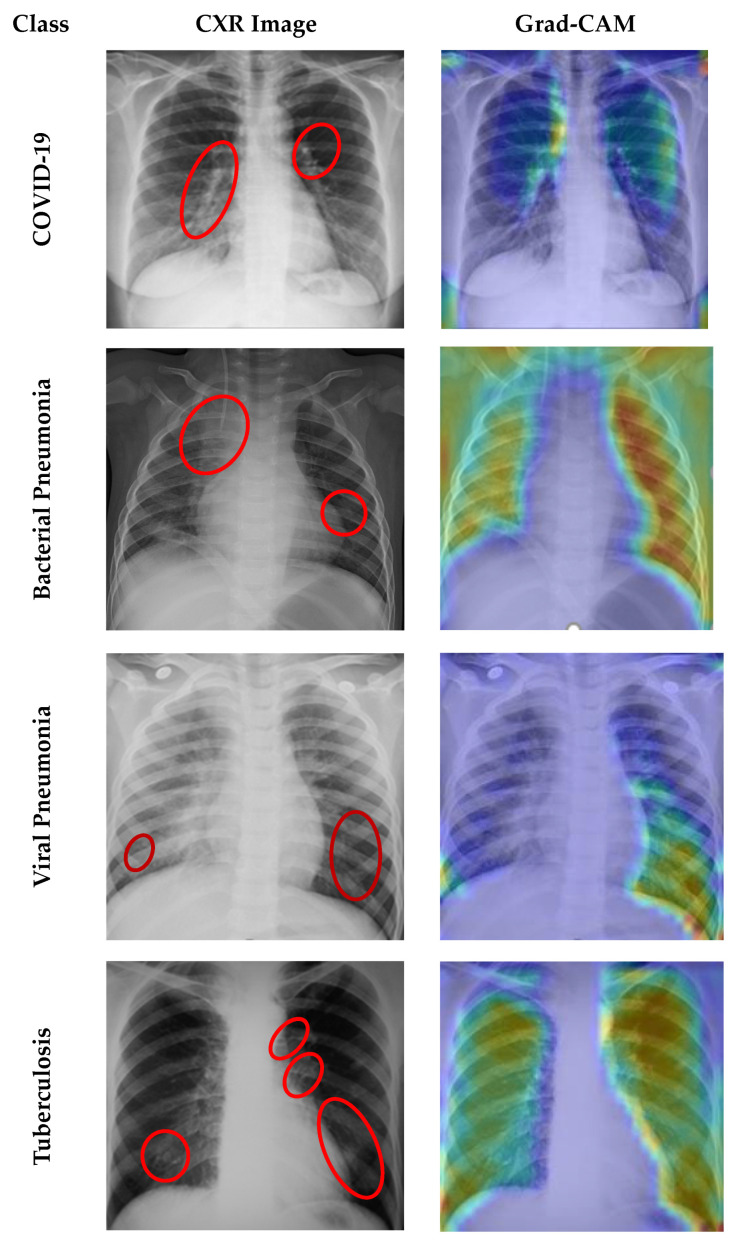

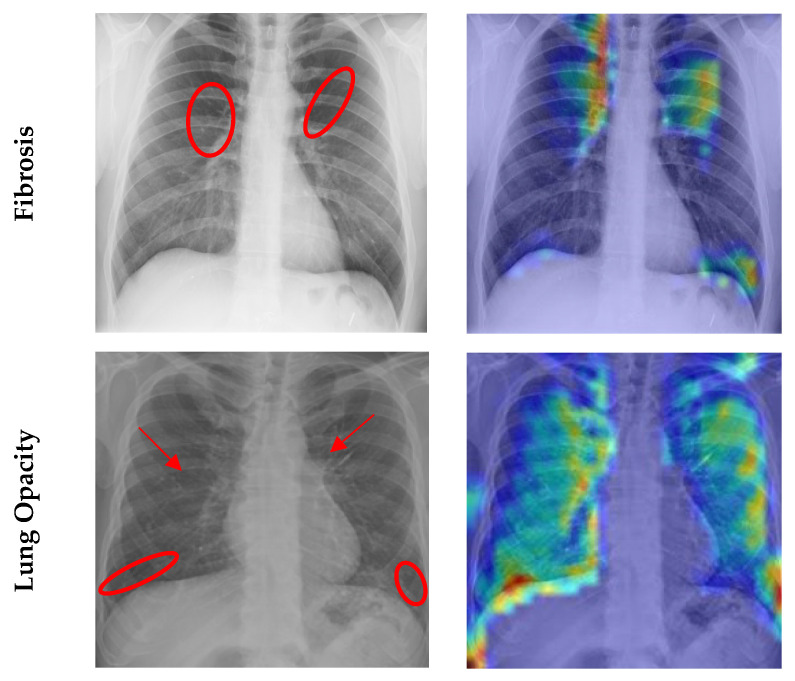
Original and Grad-CAM images of the proposed 2D-CNN model with six individual classes (COVID-19, bacterial pneumonia, viral pneumonia, tuberculosis, fibrosis, and lung opacity). Red circles and arrows indicate affected zones.

A Grad-CAM analysis highlights the activations that had the greatest contribution to predicting a given category by using gradients of the last convolution layer [42]. This study includes Grad-CAM that shows which parts of an input image have the most contribution to the proposed CNN network’s possible responses. In Figure 17, the Grad-CAM representation of the distinct six disease classes is illustrated. The original images are interpreted by radiologists as showing infected regions. As can be seen from the figure, the proposed CNN model accurately identifies the affected area for six lung diseases. Grad-CAM demonstrates that the red color denotes a region’s better significance to the model, while the blue color denotes a region’s lower priority. However, Grad-CAM produced poor localization of the heatmap in the cases of bacterial pneumonia and tuberculosis when compared to the labelling by the radiologists. Therefore, the heatmaps should be interpreted carefully.

## 5. Discussion

### 5.1. Comparative Analysis of Different Classes

**Figure 18:**
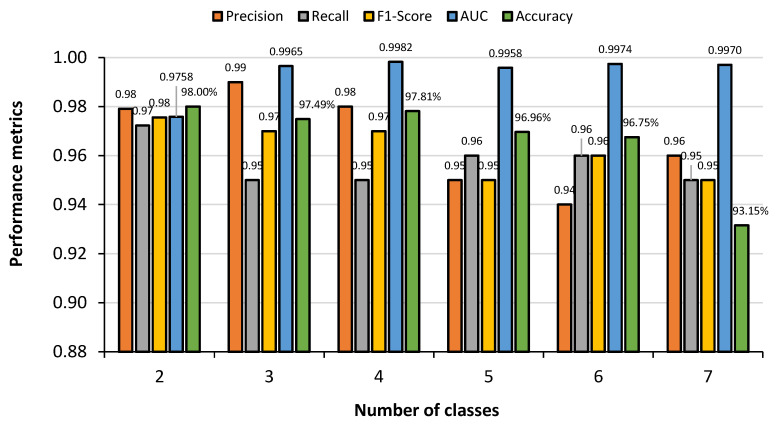
Comparison of performance metrics for binary and multiclass schemes obtained by 2-D CNN for identifying COVID-19 infection.

A comparative analysis of the proposed CNN model in accurately detecting COVID-19 for the different classification schemes considered is presented in Figure 18. It was clear from the figure that in the case of seven-class scheme, the testing accuracy was lowest (93.15%), but when the number of the class was dropped, the testing accuracy improved; for instance an accuracy of 98.00% was obtained for the binary class. However, a slight inconsistency was found between the three-class and four-class schemes. Increasing class means adding additional images in the training and testing datasets, which reduces the accuracy. Higher AUC values even at higher number of classes indicated the model has the capability of classifying COVID-19 correctly even from a large number of lung diseases. However, an anomaly is observed here. In the case of binary classification, the AUC score is found lower than the higher number of classes. This scenario happens because there are very few discriminant features in the case of binary classification. The addition of new classes means the addition of new training images. In the same way, the addition of new training images means the addition of new discriminant features. As a result, the AUC score increased when new classes are added after binary classification.

### 5.2. Comparison to Related Works in the Literature

A comparison between the proposed COVID-19 identification technique and other research performed by deep learning algorithms based on the CXR images with binary, three-class, four-class, and five-class has been presented in Table 11. Al-Waisy et al. [43] proposed the COVID-CheXNet system, a hybrid deep-learning architecture that successfully diagnosed COVID-19 patients with an accuracy rate of 99.99%. As for binary and three-class classifications, Al-Shourbaji et al. [44] proposed a batch-normalized convolutional neural network (BNCNN) model and used other pre-trained models such as VGG-16, VGG-19, Inception-V3, and ResNet-50 to detect COVID-19 and other lung diseases from the CXR images. Their results showed that the BNCNN model outperformed the pre-trained models, with accuracies of 99.27% for the binary class and 96.84% for three-class classifications, respectively.

Xu et al. [45] created an early detection system that could distinguish COVID-19 from the viral pneumonia and healthy people in a three-class classification system. The ResNet-18 model was employed and achieved an accuracy of 86.7%. The major shortcomings of the above studies were that the authors focused on two or three classes with a relatively smaller dataset. CoviXNet, a novel 15-layer CNN design presented by Srivastava et al. [46], outperformed pre-trained networks with accuracy levels of 99.47% for the binary class and 96.61% for three-class classifications, respectively.

Transfer learning techniques (VGG-19, MobileNet-v2, Inception, Xception, and Inception ResNet-v2) for detecting COVID-19 were used by Apostolopoulos et al. [47] with slightly larger number of images. This study involved 504 healthy, 700 pneumonia, and 224 recognized COVID-19 radiological images. Their top-performing model, VGG-19, had accuracy ratings of 98.75% for the binary class problem and 93.48% for the three-class problem. Yoo et al. [48] used the pre-trained ResNet18-based 2D-CNN model for classifying normal, TB, non-TB, and COVID-19. A total of 120 COVID-19 samples were collected, while 42 COVID-19 samples were tested with an average accuracy of 95%. In the study by Hussain et al. [49], a custom CNN model named “CoroDet” was proposed. The model consisted of 22 layers and was trained on images of COVID-19, normal, bacterial pneumonia, and viral pneumonia. The results showed that the model achieved an accuracy of 99.1% for two classes (COVID-19 and normal), 94.2% for three classes (COVID-19, normal, and pneumonia), and 91.2% for four classes (COVID-19, normal, bacterial pneumonia, and viral pneumonia). Khan et al. [50] introduced a deep neural network method called CoroNet trained with four classes including COVID, Normal, Bacterial Pneumonia, and Viral Pneumonia and achieving 89.6%, 95%, and 99% accuracy on two-, three-, and four-class schemes, respectively. Despite the fact that the authors found binary classification to be more accurate, three- and four-class systems were found to be less accurate. Using a combination of machine learning and deep learning techniques, Al-Timemy et al. [51] classified CXR images into either five or two classifications. A Resnet-50 deep learning model and an ensemble of subspace discriminant classifiers were utilized. The results demonstrated that the combination of these strategies yielded an accuracy of 91.60% for five class schemes and 99% for two class schemes.

Motivated by these limits, the authors of this study integrated multiple databases to build a dataset of 18,564 CXR images classified into seven classifications. In this investigation, four CNN models were applied; however, the proposed CNN model outperformed the other CNN models in discriminating COVID-19 from other lung disorders as well as healthy individuals with an accuracy of 93.15% for the seven-class schemes.

### 5.3. Strength and Limitation

The proposed CNN model can differentiate between seven distinct classes and is a robust classification model. The dataset used to train the model is a combination of three distinct datasets. As a result, the model is able to recognize disease specific distinctive features of abnormalities inside the X-ray images. The infected areas provide the essential features to the 2D-CNN model which is responsible for detection of the diseases. In addition, the model is trained and validated with two to six classes of disorders, and the experimental results have improved the model’s accuracy. The model can detect diseases in a very short period of time.

Despite these advantages, the model has several disadvantages. The performance of binary classification is somewhat subpar in comparison to contemporary research. The datasets utilized to create the fusion were not evenly distributed. It influenced the model’s performance. Despite the vast size of the dataset, it was necessary to employ a broad range of data obtained from individuals of other nationalities and countries. It will increase the model’s robustness. The model used, the parameters, and the data are all the factors that could affect the results. Further work is required to fine-tune the hyperparameters and determine whether smaller models are preferable to large pre-trained ones.

More clinical studies evaluated with clinical metrics with larger datasets are required to determine the eligibility of deploying the model in real-world situations. 

## 6. Conclusions

This work demonstrates an effective way of identifying COVID-19 from other lung diseases as well as healthy samples using the proposed CNN and three pre-trained models such as VGG-16, Inception-v3, and VGG-19. In this study, a larger dataset was constructed with 18,664 CXR images by combining three public Kaggle databases labeled with seven classes followed by data augmentation to make the classes balanced. The proposed CNN model outperformed the three pretrained models, achieving 93.15% accuracy, along with average precision, recall, f1-score, and AUC of 0.9343, 0.9443, 0.9386, and 0.9939, respectively, for the seven-class scheme. However, the other pre-trained models such as VGG16, VGG-19, and Inception-v3 also showed poorer classification accuracies of 88.33%, 90.41%, and 89.06%, respectively, for the same classification scheme. In addition, the proposed CNN model required less training and testing times. The proposed CNN model predicted the outcomes within 22.37 s, while the VGG-16, VGG-19, and Inception V3 models required 24.73 s, 27.20 s, and 42.42 s, respectively. For the COVID-19 class, the proposed model produced an AUC of 0.9970, while VGG-16, Inception-v3, and VGG-19 showed AUCs of 0.9961, 0.9929, and 0.9959, respectively, indicating the proposed model’s capability of classifying COVID-19 with high accuracy. Similarly, the proposed CNN model outperformed the pre-trained models in identifying COVID-19 for other classification schemes with testing accuracies of 98%, 97.49%, 97.81%, 96.96%, and 96.75% for two, three, four, five, and six class situations, respectively.

The future task in this research is to classify the lung diseases with a larger number of disease categories and images in the dataset using alternative deep learning algorithms. In future experiments, a validation set will be included to prove the proposed model’s reliability.

## Figures and Tables

**Table 1:** Details of the individual dataset characteristics and new dataset creation criteria.

Dataset	Image Count	Characteristics	New Dataset Inclusion Criteria
COVID-19 Radiography Database [29]	Normal: 3616 Lung Opacity: 3616 COVID-19: 3616 Viral Pneumonia: 1345	The size of each image is 299 × 299 pixels and PNG format	Each of the four class images is employed in this study.
Pneumonia Virus vs. Pneumonia Bacteria Database [30,31]	Bacterial Pneumonia: 2530 Viral Pneumonia: 1345	Images are in variable size (Max: 2008 × 2096 pixels and Min: 888 × 454 pixels) and JPEG format.	Only bacterial pneumonia images are used in this study
Chest X-Ray (CXR) images of COVID-19, Tuberculosis, Pneumonia, and Fibrosis [32,33]	COVID-19: 3616 Fibrosis: 1686 Tuberculosis: 3500 Pneumonia: 4265	Images of TB are 518 × 518 pixels in size, whereas those of fibrosis images are 1024 × 1024 pixels and both in PNG format.	Only tuberculosis and fibrosis images are utilized in this work

**Table 2:** Data distribution among different classes for training and testing.

Classes	Number of Images	Training Set	Testing Set
Normal	3616	2892	724
COVID-19	3616	2892	724
Lung Opacity	3616	2892	724
Viral Pneumonia	1345	1076	269
Bacterial Pneumonia	2530	2024	506
Tuberculosis	3500	2800	700
Fibrosis	1686	1348	338
Total	18,564	15,924	2640

**Table 3:** Summary of the proposed 2D-CNN model.

Layer (Type)	Output Shape	Parameter
conv2d_1 (Conv2D)	(None, 299, 299, 32)	896
max_pooling2d_1 (MaxPooling2D)	(None, 100, 100, 32)	0
conv2d_2 (Conv2D)	(None, 100, 100, 64)	18,496
max_pooling2d_2 (MaxPooling2D)	(None, 34, 34, 64)	0
conv2d_3 (Conv2D)	(None, 34, 34, 128)	738,560
max_pooling2d_3 (MaxPooling2D)	(None, 12, 12, 128)	0
flatten_1 (Flatten)	(None, 18,432)	0
dense_1 (Dense)	(None, 512)	9,437,696
dropout_1 (Dropout)	(None, 512)	0
dense_2 (Dense)	(None, 256)	131,328
dropout_2 (Dropout)	(None, 256)	0
dense_3 (Dense)	(None, 128)	32,896
dropout_3 (Dropout)	(None, 128)	0
dense_4 (Dense)	(None, 7)	903

**Table 4:** Number of parameters of applied models.

Model	Total Parameters	Trainable Parameters	Non-Trainable Parameters
Proposed CNN	9,696,071	9,696,071	0
VGG-16	40,412,999	25,698,311	14,714,688
VGG-19	45,722,695	25,698,311	20,024,384
Inception-v3	156,028,711	134,225,927	21,802,784

**Table 5:** Classification performance results for the seven-class scheme.

Classification Models	Images Classes	Precision	Recall	F1-Score	AUC	Testing Accuracy (%)	Average Precision	Average Recall	Average F1-Score	Average AUC	Training Time (min)	Testing Time (s)
Proposed CNN	Bacterial Pneumonia (0 *)	**0.97 ^§^**	**0.99**	**0.98**	**0.9997**	**93.15**	**0.9343**	**0.9443**	**0.9386**	**0.9939**	**62.74**	**22.37**
	COVID-19 (1)	**0.96**	0.95	**0.95**	**0.9970**							
	Fibrosis (2)	**0.99**	**0.98**	**0.98**	**0.9968**							
	Lung Opacity (3)	0.85	0.89	**0.87**	0.9848							
	Normal (4)	0.84	**0.90**	**0.87**	**0.9853**							
	Tuberculosis (5)	0.98	**0.99**	**0.99**	0.9991							
	Viral Pneumonia (6)	**0.95**	**0.91**	**0.93**	**0.9947**							
VGG-16	Bacterial Pneumonia (0)	0.92	0.91	0.91	0.9961	88.33	0.8814	0.8557	0.8614	0.9899	69.75	24.73
	COVID-19 (1)	0.92	**0.96**	0.94	0.9961							
	Fibrosis (2)	0.90	0.61	0.73	0.9843							
	Lung Opacity (3)	0.78	**0.92**	0.84	**0.9852**							
	Normal (4)	**0.88**	0.81	0.84	0.9807							
	Tuberculosis (5)	0.98	0.98	0.98	**0.9995**							
	Viral Pneumonia (6)	0.79	0.80	0.79	0.9877							
Inception-v3	Bacterial Pneumonia (0)	0.95	0.88	0.91	0.9966	89.06	0.8886	0.8729	0.8771	0.9879	71.57	42.42
	COVID-19 (1)	0.95	0.89	0.92	0.9929							
	Fibrosis (2)	0.89	0.75	0.81	0.9821							
	Lung Opacity (3)	0.82	0.90	0.86	0.9822							
	Normal (4)	0.85	0.89	0.87	0.9791							
	Tuberculosis (5)	0.97	0.98	0.97	0.9991							
	Viral Pneumonia (6)	0.79	0.82	0.80	0.9838							
VGG-19	Bacterial Pneumonia (0)	092	0.96	0.94	0.9976	90.41	0.9014	0.8929	0.8957	0.9925	76.02	27.20
	COVID-19 (1)	**0.96**	0.91	0.93	0.9959							
	Fibrosis (2)	0.84	0.84	0.84	0.9905							
	Lung Opacity (3)	**0.87**	0.88	0.87	0.9929							
	Normal (4)	0.84	0.88	0.86	0.9811							
	Tuberculosis (5)	**0.99**	0.97	0.98	0.9994							
	Viral Pneumonia (6)	0.89	0.81	0.85	0.9904							

**Note:** * The numbers 0 to 6 indicate different classes. **^§^** Bold letters indicate the best results among the models with different classes for a particular performance metric.

**Table 6:** Classification performance results for the six-class scheme.

Classification Models	Images Classes	Precision	Recall	F1-Score	AUC	Testing Accuracy (%)	Average Precision	Average Recall	Average F1-Score	Average AUC	Training Time (min)	Testing Time (s)
2D-CNN	Bacterial Pneumonia (0 *)	**0.96** **^§^**	**0.98**	**0.97**	**0.9994**	**96.75**	**0.9343**	**0.9443**	**0.9386**	**0.9939**	**62.74**	**15.20**
	COVID-19 (1)	0.97	0.96	**0.96**	0.9974							
	Fibrosis (2)	**0.99**	**0.98**	**0.99**	**0.9999**							
	Normal (3)	**0.96**	**0.97**	**0.96**	**0.9974**							
	Tuberculosis (4)	0.98	**0.99**	**0.99**	**0.9993**							
	Viral Pneumonia (5)	**0.96**	**0.90**	**0.93**	**0.9953**							
VGG-16	Bacterial Pneumonia (0)	0.83	0.83	0.83	0.9844	90.43	0.8814	0.8557	0.8614	0.9899	49.79	21.72
	COVID-19 (1)	**0.98**	0.95	0.96	**0.9978**							
	Fibrosis (2)	0.91	0.86	0.88	0.9927							
	Normal (3)	0.90	0.93	0.92	0.9910							
	Tuberculosis (4)	0.97	0.99	0.98	0.9984							
	Viral Pneumonia (5)	0.68	0.67	0.67	0.9684							
Inception-v3	Bacterial Pneumonia (0)	0.91	0.90	0.90	0.9930	91.90	0.8886	0.8729	0.8771	0.9879	51.23	18.44
	COVID-19 (1)	0.96	0.93	0.95	0.9952							
	Fibrosis (2)	0.89	0.91	0.90	0.9955							
	Normal (3)	0.93	0.91	0.92	0.9909							
	Tuberculosis (4)	0.94	0.98	0.96	0.9981							
	Viral Pneumonia (5)	0.78	0.80	0.79	0.9824							
VGG-19	Bacterial Pneumonia (0)	0.83	0.88	0.85	0.9857	89.51	0.9014	0.8929	0.8957	0.9925	53.31	24.16
	COVID-19 (1)	0.88	0.98	0.93	0.9956							
	Fibrosis (2)	0.94	0.80	0.87	0.9914							
	Normal (3)	0.91	0.90	0.90	0.9883							
	Tuberculosis (4)	**0.99**	0.96	0.97	0.9990							
	Viral Pneumonia (5)	0.73	0.62	0.67	0.9666							

**Note:** * The numbers 0 to 5 indicate different classes. **^§^** Bold letters indicate the best results among the models with different classes for a particular performance metric.

**Table 7:** Classification performance results for the five-class scheme.

Classification Model	Class	Precision	Recall	F1-Score	AUC	Testing Accuracy	Testing Time (s)
Proposed CNN	Bacterial Pneumonia (0 *)	1.00	0.99	0.99	0.9999	-	-
	COVID-19 (1)	0.95	0.96	0.95	0.9958	-	-
	Fibrosis (2)	0.96	0.99	0.98	0.9971	96.96%	16.39
	Normal (3)	0.96	0.93	0.95	0.9966	-	-
	Tuberculosis (4)	0.97	0.99	0.98	0.9990	-	-

**Note:** * The numbers 0 to 4 indicate different classes.

**Table 8:** Classification performance results for the four-class scheme.

Classification Model	Class	Precision	Recall	F1-Score	AUC	Testing Accuracy	Testing Time (s)
Proposed CNN	Bacterial Pneumonia (0 *)	0.99	1.00	1.00	1.000	97.81%	14.20
	COVID-19 (1)	0.98	0.95	0.97	0.9982		
	Normal (2)	0.97	0.98	0.97	0.9986		
	Tuberculosis (3)	0.98	0.99	0.98	0.9984		

**Note:** * The numbers 0 to 3 indicate different classes.

**Table 9:** Classification performance results for the three-class scheme.

Classification Model	Class	Precision	Recall	F1-Score	AUC	Testing Accuracy	Testing Time (s)
2D-CNN	Bacterial Pneumonia (0 *)	0.99	0.99	0.99	0.9997	97.49%	6.73
	COVID-19 (1)	0.99	0.95	0.97	0.9965		
	Normal (2)	0.95	0.99	0.97	0.9997		

**Note:** * The numbers 0 to 2 indicate different classes.

**Table 10:** Classification performance results for the two-class scheme.

Classification Model	Class	Precision	Recall	F1-Score	AUC	Testing Accuracy	Testing Time (s)
2D-CNN	Normal	0.9791	0.9723	0.9756	0.9758	98%	6
	COVID-19						

**Table 11:** Comparison between the proposed COVID-19 diagnostic approaches and related works.

Research	Classes	Image Count	Model Applied	Results
Al-Waisy et al. [43]	2	COVID-19 = 400 and Normal = 400	COVID-CheXNet	Accuracy = 99.99%
Al-Shourbaji et al. [44]	2	COVID-19 = 3616 and Normal = 10192	BNCNN	Accuracy = 99.27%
Xu et al. [45]	3	COVID-19 = 219 Viral pneumonia = 224 Healthy = 175	ResNet-18	Accuracy= 86.7%
Srivastava et al. [46]	2	COVID-19 = 1281 Normal = 3270 viral-pneumonia = 1656	CoviXNet	Accuracy = 99.47%
	3			Accuracy = 96.61%
Apostolopoulos et al. [47]	2	COVID-19 = 224 Pneumonia = 700 Healthy = 504	VGG-19	Accuracy = 98.75%
	3			Accuracy = 93.48%
Yoo et al. [48]	4	Normal = 120 TB = 120 Non-TB = 120 COVID-19 =120	ResNet18	Average Accuracy = 95%
Hussain et al. [49]	2	COVID-19 = 500 Normal = 800 Viral pneumonia = 400 Bacterial Pneumonia = 400	CoroDet	Accuracy = 99.1%
	3			Accuracy = 94.2%
	4			Accuracy = 91.2%
Khan et al. [50]	2	COVID-19 = 290 Normal = 1203 Viral pneumonia = 931 Bacterial Pneumonia = 660	CoreNet	Accuracy = 99%
	3			Accuracy = 95%
	4			Accuracy = 89.6%
Al-Timemy et al. [51]	2	Normal = 439 COVID-19 = 435 Bacterial Pneumonia = 439 Viral Pneumonia = 439 TB = 434	Resnet-50 with ensemble of subspace discriminant classifier	Accuracy = 99%
	5			Accuracy = 91.60%
Proposed Work	2	COVID-19 = 3616 Normal = 3616	2D-CNN	Accuracy: 2D-CNN = 98%
	3	COVID-19 = 3616 Normal = 3616 Bacterial Pneumonia = 2530	2D-CNN	Accuracy: 2D-CNN = 97.49%
	4	COVID-19 = 3616 Normal = 3616 Tuberculosis = 3500 Bacterial Pneumonia = 2530	2D-CNN	Accuracy: 2D-CNN = 97.81%
	5	COVID-19 = 3616 Normal = 3616 Fibrosis = 1686 Bacterial Pneumonia = 2530 Tuberculosis = 3500	2D-CNN	Accuracy: 2D-CNN = 96.96%
	6	COVID-19 = 3616 Normal = 3616 Fibrosis = 1686 Viral Pneumonia = 1345 Bacterial Pneumonia = 2530 Tuberculosis = 3500	2D-CNN, VGG-16, Inception-v3, and VGG-19	Accuracy: 2D-CNN = 96.75% VGG-16 = 90.43% VGG-19 = 89.51% Inception-v3 = 91.90%
	7	COVID-19 = 3616 Normal = 3616 Lung Opacity = 3616 Viral Pneumonia = 1345 Bacterial Pneumonia = 2530 Tuberculosis = 3500 Fibrosis = 1686	2D-CNN, VGG-16, Inception-v3, and VGG-19	Accuracy: 2D-CNN = 93.15% VGG-16 = 88.33% VGG-19 = 90.41% Inception-v3 = 89.06%

## Data Availability

Not applicable.
